# Role of Yb^3+^ ions on enhanced ~2.9 μm emission from Ho^3+^ ions in low phonon oxide glass system

**DOI:** 10.1038/srep29203

**Published:** 2016-07-04

**Authors:** Sathravada Balaji, Gaurav Gupta, Kaushik Biswas, Debarati Ghosh, Kalyandurg Annapurna

**Affiliations:** 1 Glass Science and Technology Section, CSIR-Central Glass and Ceramic Research Institute, 196, Raja S. C. Mullick Road, Kolkata–700 032, India

## Abstract

The foremost limitation of an oxide based crystal or glass host to demonstrate mid- infrared emissions is its high phonon energy. It is very difficult to obtain radiative mid-infrared emissions from these hosts which normally relax non-radiatively between closely spaced energy levels of dopant rare earth ions. In this study, an intense mid-infrared emission around 2.9 μm has been perceived from Ho^3+^ ions in Yb^3+^/Ho^3+^ co-doped oxide based tellurite glass system. This emission intensity has increased many folds upon Yb^3+^: 985 nm excitation compared to direct Ho^3+^ excitations due to efficient excited state resonant energy transfer through Yb^3+^: ^2^F_5/2_ → Ho^3+^: ^5^I_5_ levels. The effective bandwidth (FWHM) and cross-section (σ_em_) of measured emission at 2.9 μm are assessed to be 180 nm and 9.1 × 10^−21^ cm^2^ respectively which are comparable to other crystal/glass hosts and even better than ZBLAN fluoride glass host. Hence, this Ho^3+^/Yb^3+^ co-doped oxide glass system has immense potential for the development of solid state mid-infrared laser sources operating at 2.9 μm region.

Laser materials operating in the 2–5 μm region are gaining much interest in recent years because of their wide applications in automotive, pharmaceutical and medical industries^1^,^2^. This wavelength region being known as “finger print region” for many molecules, lasers operating at this region can potentially act as chemical sensors too^3^. Especially, Mid Infrared (MIR) photonics has gained momentum ever since the invention of quantum cascade lasers^4^ which are currently being served as laser sources in MIR region. However, because of their miniature size, heat dissipation has become a main threat where, around 70% of input pump power has been obsessive only for heat generation^5^. On the other hand optical parametric oscillator (OPO) which also being served as MIR laser sources are expensive and require highly coherent pump sources^6^. In recent years, significant thrust has been driven on rare earth doped low phonon fluoride and chalcogenide^1^,^6^,^7^ based glasses because of their flexible geometry and easy fiberization than that of crystals. Although fluoride glasses are thermally more stable than chalcogenide glasses, chemical durability and mechanical strength of these glasses are inferior to that of oxide glasses. Chalcogenide glasses on the other hand, having high transparency far beyond fluoride glasses, and can potentially allow many MIR emission transition from rare earth ions. Conversely, high refractive index of these materials may restrict high peak powers of MIR emissions. Further, preparation process for fluoride and chalcogenide based glasses are onerous compared to oxide glasses. Hence, rare earth doped oxide based glasses with low phonon energy and extended infrared transmission are attaining high surge to serve as MIR laser sources provided the glasses should have zero/low OH^−^ content. In this contest, tellurite glasses (transparency upto ~6 μm) have been under strenuous study because of their advantages over fluoride and chalcogenide based glasses. There are few reports on ~2.7 μm emission from Er^3+^ ions^8^,^9^,^10^, ~2.8 μm, ~4 μm from Ho^3+^ ions^11^, ^12^, ^13^, ^14^ and ~2.9 μm, ~3.3 μm from Dy^3+^ ions^15^,^16^ when doped in tellurite glasses. However, the reported ~4 μm emission from Ho^3+^ ions is subject to revision since harmonic peak of Ho^3+^: ~2 μm emission arises exactly at ~4 μm and it is very difficult to eliminate this harmonic peak unless one suppresses the ~2 μm emission by using a proper high pass IR cut-on filter >2 μm. Also, there is no mention of filters used while recording the ~4 μm MIR emission in their report^13^,^14^.

In preview of Ho^3+^ ion energy level structure, it can facilitate numerous NIR and MIR emission transitions when doped into a suitable low phonon host material^6^. However, one of the major shortcomings of Ho^3+^ is the lack of ground state absorption (GSA) transitions^17^ that overlap with convenient high-power pump sources. Thus, sensitized excitation with suitable rare earth ion co-doping having strong absorption at pump powers seems to be a better choice. Earlier, we have demonstrated^18^ energy transfer (Yb^3+^ → Ho^3+^) based enhanced and efficient ~2 μm emission from Ho^3+^/Yb^3+^ ions co-doped Tellurium-Barium-Lanthanum oxide glass system having low phonon energy. Also, visible up-conversion emissions from Ho^3+^ ions on account of bi-photonic absorption by Yb^3+^ ions under 980 nm excitation in the same host has been reported^19^. The present work mainly aims to investigate the MIR emission transitions from Ho^3+^ ions by considering the extended IR transparency (upto ~6 μm) and low phonon energy (Fig. 1 of Supplementary data) of this oxide based glass system. The dependence of emission transition strengths on pump wavelengths, role of Yb^3+^ co-doping on the enhancement of Ho^3+^ mid-infrared emissions and the possible energy transfer mechanisms were discussed in detail and reported here.

## Results and Discussion

**Figure 1:**
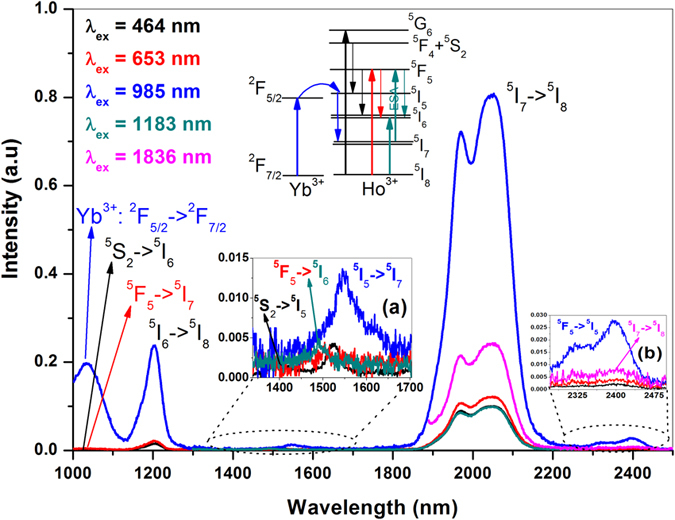
NIR emission spectra of Yb^3+^/Ho^3+^ co-doped glass under different excitation wavelengths (colour codes were given for different excitation wavelengths).

The emission spectra in NIR (1.0–2.5 μm) and MIR (2.5 to 4.5 μm) regions were recorded at room temperature under similar experimental conditions using suitable combinations of low pass and high pass filters (Fig. 2 of Supplementary data) at excitation and emission channels respectively. Figure 1 presents the NIR emission spectra of co-doped sample recorded by exciting Yb^3+^ (985 nm) and Ho^3+^ (464 nm, 653 nm, 1183 nm and 1836 nm) ions corresponding to their intense absorption transitions. The spectra have revealed characteristic emission bands from Yb^3+^ and Ho^3+^ ions which are designated to appropriate transitions depending on their energy positions. Further, there is a clear dependence of excitation wavelength on emission intensity where an ~8 fold enhancement in ~2 μm emission has been demonstrated under Yb^3+^ (985 nm)excitation compared to direct Ho^3+^ ion excitations (except 1836 nm excitation, where only ~4 fold enhancement was observed). This has been attributed to an effective energy transfer from Yb^3+^ to Ho^3+^ ions with estimated transfer efficiency of 86% (ref. 18).

The insets (a & b) of Fig. 1 show the magnified view of emission transitions in respective wavelength regions along with partial energy level diagram projecting those emission transitions. The spectra shown in inset (a) of Fig. 1 (1300–1700 nm region) appears to be greatly dependent on the excitation wavelength. Under Ho^3+^: 464 nm excitation, two emission bands observed at 1400 nm and 1530 nm are assigned to the transitions ^5^S_2_ → ^5^I_5_ and ^5^F_5_ → ^5^I_6_ respectively. But, the Ho^3+^: 653 nm and 1183 nm excitations show only ^5^F_5_ → ^5^I_6_ emission transition slightly at higher energy side ~1490 nm. In case of 1183 nm excitation, this emission is normally not anticipated due to huge energy level mismatch. However, the experimental emission data suggests, this emission transition is only possible with second photon absorption from ^5^I_7_ to ^5^F_5_ state through Excited State Absorption (ESA) process. The calculated excited state lifetime of ^5^I_7_ state is in the order of ~5 ms (radiative lifetime calculated through J-O analysis, Table 1 of Supplementary data)^18^ clearly indicates the probability of ESA from ^5^I_7_ → ^5^F_5_ is at higher side. Interestingly, under Yb^3+^: 985 nm excitation an enhancement in intensity of emission at ~1550 nm is observed with slight red shift in its peak. Based on the energy level position of Ho^3+^ ions, this emission peak is assigned to ^5^I_5_ → ^5^I_7_ transition^20^. As discussed above, the Ho^3+^: ~1550 nm emission can originate from ^5^F_5_, ^5^I_5_ levels, to ascertain the originating state probability, the radiative transition rate calculated from J-O analysis have been considered as an effective tool. The emission probability (from J-O analysis) for ~1550 nm originating from Ho^3+^: ^5^I_5_ level is ~65% (Table 1 of Supplementary data), whereas the ~1490/~1530 nm emission originating from Ho^3+^: ^5^F_5_ level is only 4% clearly indicates the prominence of ^5^I_5_ → ^5^I_7_ transition over ^5^F_5_ → ^5^I_6_. Further, upon Yb^3+^: 985 nm excitation the ^5^I_5_ level is effectively populated through resonant energy transfer process which enhances the transition probability of Ho^3+^: ^5^I_5_ → ^5^I_7_ (~1550 nm) over Ho^3+^: ^5^F_5_ → ^5^I_6_ transition.

**Figure 2:**
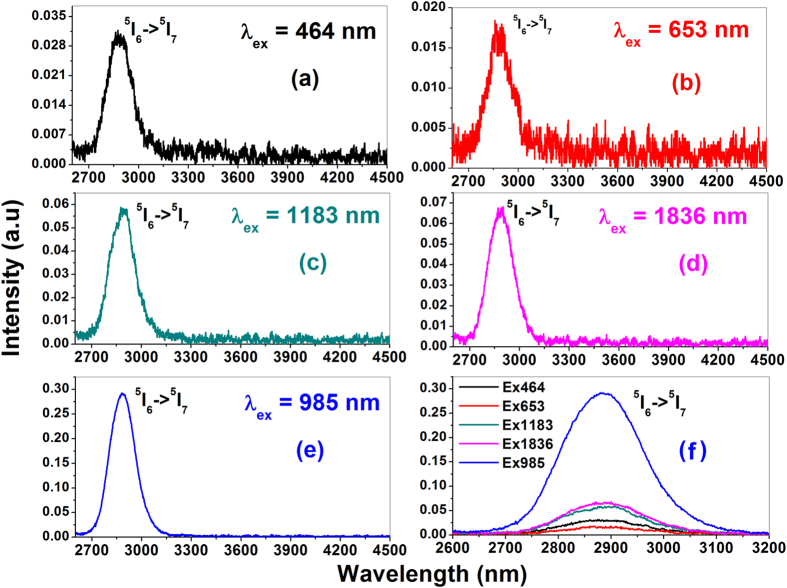
MIR emission spectra of Yb^3+^/Ho^3+^ co-doped glass under different excitation wavelengths.

**Figure 3:**
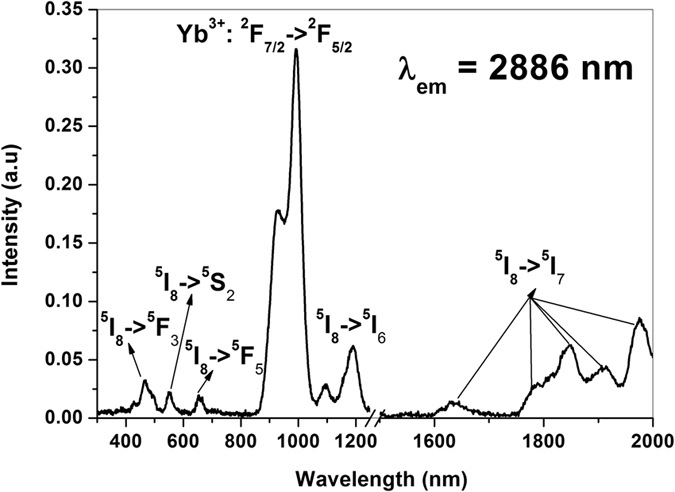
Excitation spectrum of Yb^3+^/Ho^3+^ co-doped glass monitoring 2886 nm emission of Ho^3+^ ions.

The MIR emission spectra of Ho^3+^ ions recorded under different excitation wavelengths are shown in Fig. 2(a–f). The spectra revealed an intense emission band peaking at around 2886 nm corresponding to Ho^3+^: ^5^I_6_ → ^5^I_7_ intra-manifold transition under all considered excitation wavelengths. The intensity of the band greatly depends on the excitation wavelength as shown in Fig. 2(f). Interestingly, compared to direct excitations of Ho^3+^ ions, Yb^3+^ sensitized excitation (985 nm) yielded a remarkable enhancement in the emission peak intensity. Since, the high absorption cross section (~1.9 × 10^–20^ cm^2^) of Yb^3+^ ions at 985 nm practically enhances the ^2^F_5/2_ excited state population density which favours an efficient energy transfer from Yb^3+^ to Ho^3+^ ions in the present host^18^. The MIR emission intensity has increased to ~15 folds, ~8 folds, ~4 folds and ~5 folds compared to direct Ho^3+^: 653, 464, 1183 and 1836 nm excitations respectively. The excitation spectrum as shown in Fig. 3 recorded for 2886 nm emission depicts several well resolved absorption bands of Ho^3+^ ions having lower intensity than that of Yb^3+^ ions specifies the significance of Yb^3+^ sensitization in the present host. The emission cross-section of ^5^I_6_ → ^5^I_7_ transition (at ~2.9 μm) calculated using Fuchtbauer-Ladenburg equation is found to be 9.068 × 10^–21^ cm^2^ (Fig. 3 of Supplementary data). Additionally, the full width at half maximum (FWHM) of this emission transition is 180 nm which is quite high compare to other tellurite glass hosts reported in the literature^11^,^12^. The high band width is very much useful for ultra-broadband tunable laser sources. Table 1 compares the MIR emission properties of this glass with different crystal/glass hosts reported in the literature^11^,^21^,^22^,^23^,^24^,^25^,^26^. The emission cross-section value is comparable to other hosts and even better than ZBLAN fluoride glass.

### Energy transfer mechanism

**Figure 4:**
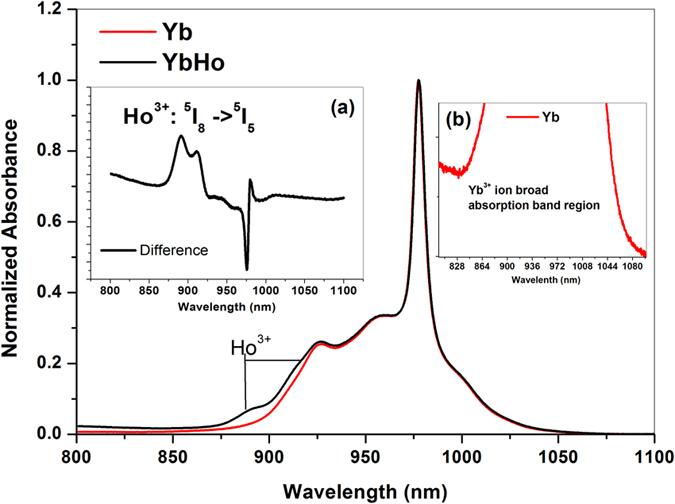
Slow scanned absorption spectra of Yb^3+^ doped and Yb^3+^/Ho^3+^co-doped glass. Inset (**a**): difference absorption spectrum; Inset (**b**): magnified view of Yb^3+^ absorption band.

The probable energy transfer mechanism contributing to this intense MIR emission at ~2.9 μm may be through resonant energy transfer from Yb^3+^: ^2^F_5/2_(highest Stark component) → Ho^3+^: ^5^I_5_ alongside the phonon assisted Yb^3+^: ^2^F_5/2_ → Ho^3+^: ^5^I_6_ energy transfer process. Since the strong absorption of Yb^3+^ ions suppresses the weak absorption transition, ^5^I_8_ → ^5^I_5_ of Ho^3+^ ions in the region 850–930 nm, and in order to elucidate the resonant energy transfer mechanism, absorption spectra of both Yb^3+^ singly doped and Yb^3+^/Ho^3+^ co-doped samples were measured with slow scan rate as shown in Fig. 4.

The absorption spectrum of co-doped glass in the higher energy side (850–930 nm) indicates the superposition of Ho^3+^: ^5^I_8_ → ^5^I_5_ absorption band which could be clearly visualized in the difference spectrum shown in the inset (a) of Fig. 4. The broadness of absorption band of Yb^3+^ ions is shown in magnified view starting from ~820 to 1100 nm in the inset (b) of Fig. 4 emphasising the greater overlap of Yb^3+^: ^2^F_5/2_ and Ho^3+^: ^5^I_5_ energy levels. Further, the mechanism of resonant energy transfer from Yb^3+^: ^2^F_5/2_ → Ho^3+^: ^5^I_5_ also be supported by the experimentally detected NIR emission spectrum where the enhanced emission at ~1550 nm (Ho^3+^: ^5^I_5_ → ^5^I_7_) has been observed in case of Yb/Ho co-doped sample which is originating from ^5^I_5_ level under 985 nm excitation.

**Figure 5:**
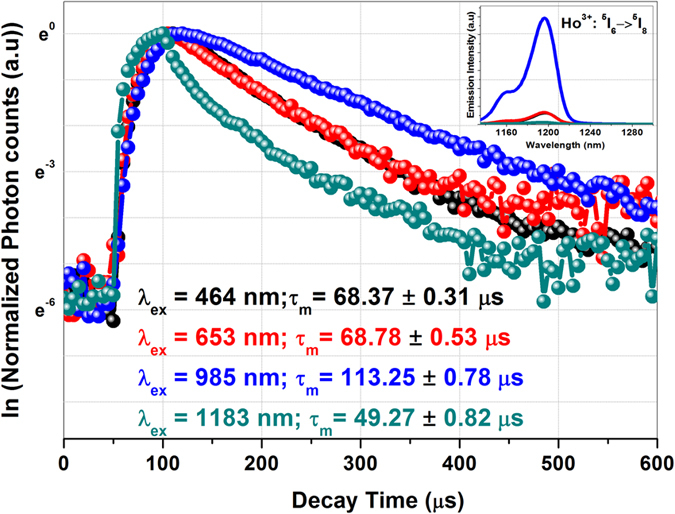
Decay dynamics of Ho^3+^: ^5^I_6_ excited state. Inset: Ho^3+^: ^5^I_6_ → ^5^I_8_ emission intensity variation with different excitation wavelengths.

To understand the dynamics of the MIR emission originating state i.e., Ho^3+^: ^5^I_6_, the decay rates were studied under different excitation wavelengths by monitoring the intense emission from ^5^I_6_ level i.e., 1.2 μm. Figure 5 presents the decay curves of the NIR emission at 1.2 μm corresponding to Ho^3+^: ^5^I_6_ → ^5^I_8_ transition under different excitation wavelengths. The decay curves were well fitted to single exponential function and the lifetime values are presented in Fig. 5 (as measured decay curves of ^5^I_6_ level under different excitations are shown in Fig. 4 of Supplementary data). Considering the fact that, the lifetime of the ions in the excited state has less influence on the different emission transitions originating particularly from the same excited state. If we see the radiative transition probability rate (calculated from J-O analysis; Table 1 of Supplementary date) of the different emission transitions originating from ^5^I_6_ level, 80.5 % of Ho^3+^ ions were contributing for NIR 1.2 μm emission and 10.5% for MIR ~2.9 μm emission. Interestingly upon Yb^3+^ excitation, the decay rate of ^5^I_6_ level has been enhanced to ~1.7 times (compared to Ho^3+^: 464 and 653 nm excitation) and ~2.3 times (compared to Ho^3+^: 1183 nm excitation). The slow feeding of excited ions from Yb^3+^: ^2^F_5/2_ (lifetime of Yb^3+^ ions in Yb^3+^ singly doped glass is ~0.63 ms where as in Yb^3+^/Ho^3+^ co-doped sample it is 0.09 ms (ref. 18)) to Ho^3+^: ^5^I_5_ state through resonant energy transfer mechanism is contributing for the excessive population at Ho^3+^: ^5^I_6_ state which inturn enhanced its decay rate upon Yb^3+^ excitation.

**Figure 6:**
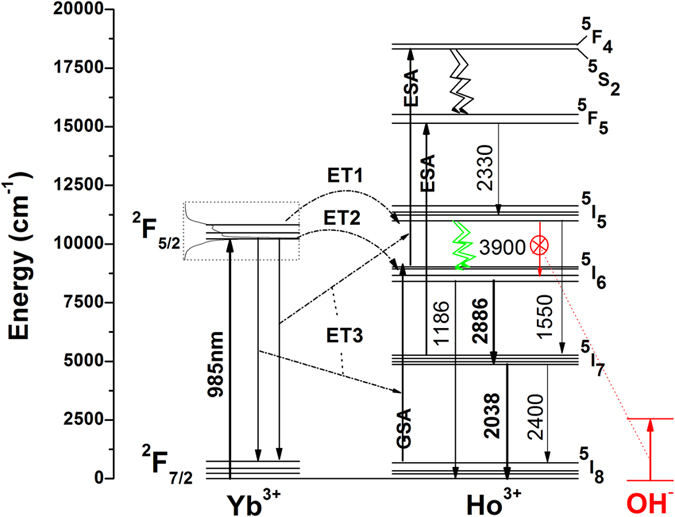
Partial Energy level diagrams of Yb^3+^ and Ho^3+^ ions depicting experimentally observed NIR and MIR emission transitions along with possible energy transfer mechanisms in the present glass system.

The partial energy level diagram illustrated in Fig. 6 presents the experimentally observed NIR and MIR emission transitions with possible Yb^3+^ → Ho^3+^ energy transfer mechanisms in the present host. In presence of Yb^3+^ ions, the energy transfer (Yb^3+^ → Ho^3+^) may take place mainly in three routes ET1, ET2 and ET3 as indicated in Fig. 6. From the observed emission intensities and their calculated emission transition strengths, probability of energy transfer mechanism may follow the trend ET1 > ET2 > ET3. The energy transfer micro parameter (C_DA_) has been calculated using the spectral overlap method. For ET1 process, the acceptor, Ho^3+^ ions absorption band ^5^I_8_ → ^5^I_5_ (peaking at ~900 nm) has been considered. For ET2 process, Ho^3+^ ions absorption band ^5^I_8_ → ^5^I_6_ (peaking at ~1200 nm) has been considered. In both the cases, donor Yb^3+^ ions emission cross-section calculated from Reciprocity Method (RM) has been considered^19^ for calculating the C_DA_ values. The obtained values are 6.05623 × 10^−33^ cm^6^ s^−1^ for ET1 process and 1.38 × 10^−40^ cm^6^ s^−1^ for ET2 process. The C_DA_ values strongly suggest that ET1 is more prominent than ET2. ET1 is resonant energy transfer process where as ET2 is phonon assisted energy transfers process. ET3 is mainly based on Excited State Absorption (ESA) process which depends on rare earth ion concentration, excited state (Ho^3+^: ^5^I_6_, ^5^I_7_) lifetime and pump power density. Under direct Ho^3+^ excitations at 464 nm and 653 nm, it follows multiple relaxations from different excited states to yield a less intense MIR emission transitions. Ho^3+^: 1183 and 1836 nm excitations show a considerable intense MIR emission transitions, again these transitions are dependent on the population densities at ^5^I_7_ level which is always a less probable route. Though a strong resonant energy transfer ET1 process (Yb^3+^: ^2^F_5/2_ → Ho^3+^: ^5^I_5_) observed in the present low phonon oxide glass system, emission at ~4 μm could not be perceived due to the low radiative transition probability (4%) or strong interaction with OH^−^ions (Fig. 5 of Supplementary data) in the glass matrix.

## Conclusions

An intense and broad (FWHM = 180 nm) MIR emission peaking at around 2.9 μm (^5^I_6_ → ^5^I_7_) from Ho^3+^ ions with a cross-section (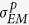) of 9.1 × 10^−21^ cm^2^ has been reported in a Yb^3+^/Ho^3+^ co-doped low phonon tellurium oxide based glass system. MIR emission intensity increased to many folds upon Yb^3+^ excitation at 985 nm compared to direct Ho^3+^ ion excitations which has been attributed to high absorption cross-section at pump wavelength followed by resonant energy transfer from Yb^3+^ → Ho^3+^ ions. The decay dynamics indicate Yb^3+^ sensitized excitation effectively enhance the Ho^3+^: ^5^I_6_ level decay time by increasing the population density through Yb^3+^: ^2^F_5/2_ → Ho^3+^: ^5^I_5_ resonant energy transfer process. The hassle free fabrication of these oxide glasses along with ease of fiberization make them potential candidates for the development of solid state MIR laser sources compatible to the commercially available high energy pump sources.

## Materials and Methods

The Ho^3+^/Yb^3+^ co-doped tellurite glass of composition (mol %) 80TeO_2_-15(BaF_2_ + BaO)-3La_2_O_3_-1Ho_2_O_3_-1Yb_2_O_3_ and only Yb^3+^ doped glass (mol %) 80TeO_2_-15(BaF_2_ + BaO)-4La_2_O_3_-1Yb_2_O_3_ were prepared by melt quenching technique. The raw chemicals used such as TeO_2_, BaF_2_, BaCO_3_, La_2_O_3_, Ho_2_O_3_ and Yb_2_O_3_ were of GR grade as procured from Sigma-Aldrich, USA with ≥99.99% purity. Each batch to yield approximately of 10 g glass was melted for about an hour in a pure platinum crucible at 700−750 ^o^C in an electrical furnace. The molten batch was stirred intermediately using a thin platinum rod to attain bubble free and homogeneous melt. The cast glasses were annealed for about 2 hours at the temperature close to glass transition range to avoid thermal stress in the glass. For optical measurements, the samples were cut and polished to a plate-shape.

The absorption spectra of the samples were recorded using UV-VIS-NIR absorption spectrophotometer (Model: Lamda950, Perkin Elmer, USA). The emission and excitation spectra of the sample were recorded at room temperature on spectrofluorimeter (Model: Quantum Master enhanced NIR from PTI, USA) fitted with double monochromators on both excitation and emission channels. The NIR (1–2.5 μm) and MIR (2.5–5 μm) emission spectra of the sample were recorded through Peltier cooled InGaS solid state detector (Model: J23TE2-66C-R02M-2.4, Judson technologies, USA) and LN_2_ cooled InSb detector (Model: P7751-02, Hamamatsu, Japan) respectively. The emission channels were equipped with 2000 nm and 4000 nm blazed gratings for InGaS and InSb detectors respectively. Appropriate low-pass and high-pass filters from Edmund Optics, Inc, USA were used at excitation and emission channels to avoid excitation and emission wavelength’s higher order harmonics in the recorded emission spectrum. The transmission spectra of the used filters were given in the Supplementary file for ready reference. The decay kinetics of Ho^3+^: ^5^I_6_ excited state were measured under different excitation wavelengths (464, 653, 985 & 1183 nm) by monitoring 1.2 μm emission corresponding to ^5^I_6_ → ^5^I_8_ transition using 60 W Xenon flash lamp as source and LN_2_ cooled NIR PMT as detector in gated mode (Model: NIR PMT 1.7 from Hamamatsu, Japan) on same spectrofluorimeter.

## Additional Information

**How to cite this article**: Balaji, S. *et al.* Role of Yb^3+^ ions on enhanced ~2.9 µm emission from Ho^3+^ ions in low phonon oxide glass system. *Sci. Rep.* **6**, 29203; doi: 10.1038/srep29203 (2016).

## Supplementary Material

Supplementary Information

## Figures and Tables

**Table 1:** MIR emission properties comparison with other host materials.

Host	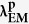 ~2.9 μm Δλ_FWHM_(nm)	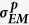 (cm^2^) 10^−20^	Reference
LLF^C^	~85	1.9	[21]
PbF_2_^C^	~120	1.4	[22]
GGG^C^	~130	0.3	[23]
Yb-Ho-YAG^C^	~50	1.2	[24]
TZNF60^G^	80	1.51	[11]
Fluoroaluminate^G^	~60	1.91	[25]
ZBLAN^G^	5	0.5	[26]
TBL^G^	180	0.91	Present Work

G: Glass; C: Crystal.
