# Supplementary material for: Role of Yb3+ ions on enhanced ~2.9 μm emission from Ho3+ ions in low phonon oxide glass system
Source: Sci Rep. 2016 Jul 4;6:29203. doi: 10.1038/srep29203 (PMC4931574; doi:10.1038/srep29203)
Supplement: Supplementary Information [file srep29203-s1.pdf]

SUPPLIMENTARY DATA:

Role of Yb^{3+} ions on enhanced ~2.9 μm emission from Ho^{3+} ions in low phonon oxide glass system

Sathravada Balaji[#], Gaurav Gupta, Kaushik Biswas, Debarati Ghosh, and Kalyandurg Annapurna[*]

Glass Science and Technology Section

CSIR-Central Glass and Ceramic Research Institute

196, Raja S. C. Mullick Road, Kolkata – 700 032, INDIA

Corresponding author email: annapurnak@cgcri.res.in[*], sbalaji@cgcri.res.in[#]

Telephone: +91-33-2322 3344/3421; Fax: +91-33-2473 0957

Table 1: Radiative properties

Ems-Trans	cm	S_{ed}	S_{md}	A_{ed}	A_{md}	A_{rad}	ΣA_{rad}	τ_{rad} (ms)	β
$^5I_7$ -->$^5I_8$	1.94E-04	7.263E-20	6.96E-21	325.8	31.4	357.2	357.2	2.8	1
$^5I_6$ -->$^5I_7$	2.90E-04	5.017E-20	0	74.41	0	74.41			0.105
-->$^5I_8$	1.19E-04	3.102E-20	0	631	0	631	705.4	1.42	0.895
$^5I_5$ -->$^5I_6$	4.09E-04	4.015E-20	0	22.96	0	22.96			0.039
$^5I_7$	1.55E-04	3.695E-20	0	382.4	0	382.4			0.646
$^5I_8$	9.00E-05	4.387E-21	0	209.3	0	209.3	591.7	1.69	0.354
$^5F_5$ -->$^5I_5$	2.33E-04	9.332E-21	0	37.12	0	37.12			0.004
$^5I_6$	1.49E-04	2.954E-20	0	368.7	0	368.7			0.041
$^5I_7$	1.03E-04	4.264E-20	0	1375	0	1375			0.154
$^5I_8$	6.47E-05	5.26E-20	0	7124	0	7124	8905	0.11	0.8
$^5F_4$, $^5S_2$--> $^5F_5$	3.20E-04	3.158E-20	0	46.94	0	46.94			0.004
$^5I_5$	1.40E-04	2.785E-20	0	525.7	0	525.7			0.039
$^5I_6$	1.03E-04	2.505E-20	0	1028	0	1028			0.077
$^5I_7$	7.60E-05	1.53E-20	0	1407	0	1407			0.105
$^5I_8$	5.50E-05	4.48E-20	0	10327	0	10327	13335	0.075	0.774

S_{ed} = Electric dipole induced emission line strength

S_{md} = Magnetic dipole induced emission line strength

A_{ed} = Electric dipole induced Spontaneous emission probability

A_{md} = Magnetic dipole induced Spontaneous emission probability

A_{rd} = A_{ed} + A_{md}

ΣA_{rad} = Total spontaneous emission probability of the state

τ_{rad} =Radiative lifetime

β = Branching ration

Figure 1: Raman and FTIR-Reflectance Spectra of Yb^{3+}/Ho^{3+} co-doped sample

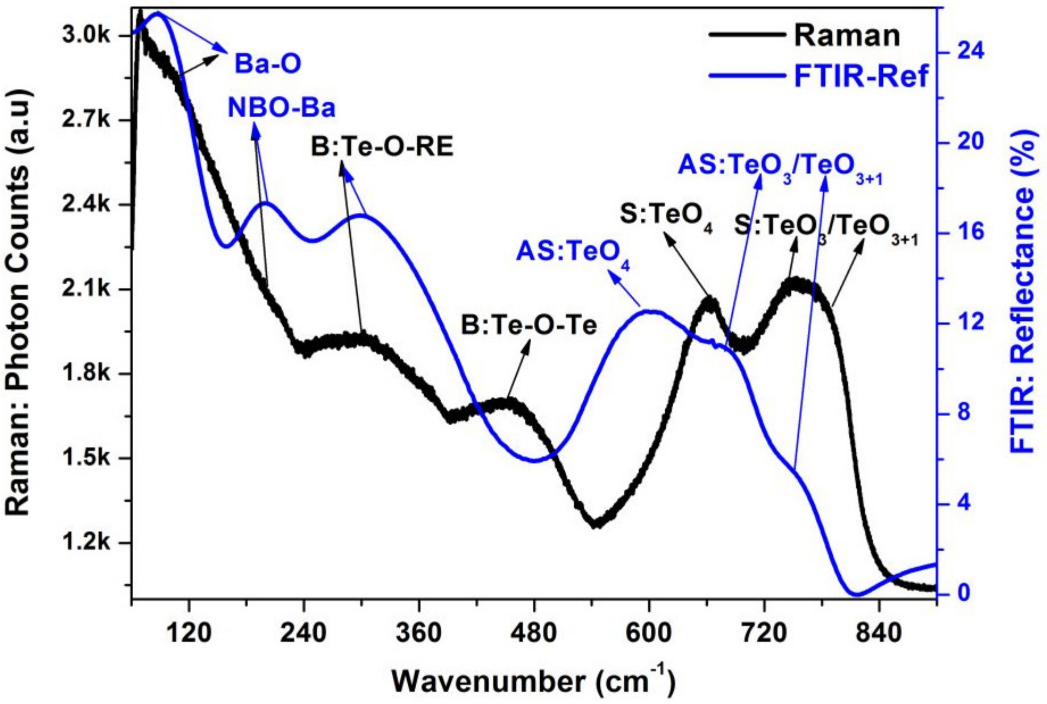

B: Bending

AS: Asymmetric

S: Symmetric

Ba-O: Barium (Metal ion) –Oxygen

NBO-Ba: Non Bridging Oxygen –Barium (Metal ion)

Note: Only for comparison, FTIR-Reflectance and Raman spectra were plotted using double Y-axis scale. The intensities of the bands have their usual meaning as shown in the graph scale.

Raman Spectrum of the glass has been recorded using 486 nm Argon ion laser on confocal Laser Raman Spectrometer (Model: Lab Ram HR 800 EV, HORIBA Jobin Yvon, France). The FTIR reflectance spectrum of the glass sample recorded using a FTIR spectrometer (Model: Frontier, FIR-MIR-FTIR, Perkin-Elmer, USA) at a 15° angle of incidence.

Raman spectrum shows distinct vibrational bands due to symmetric stretching modes of $TeO_4/TeO_3/TeO_{3+1}$in the range 600- 800 cm^{-1}, bending modes due to Te-O-Te at ~450 cm^{-1} and Te-O-RE (RE- Rare Earth element) at ~300 cm^{-1} respectively. FTIR-Reflectance spectrum as shown in the Fig 1 depicts the vibrational bands corresponding to the asymmetric stretching vibrations in the region 480- 800 cm^{-1} and

bending vibrations in the range less than 480 cm^{-1}. The vibrational bands in the lower wavenumber region of Raman as well as FTIR were assigned to the metal ion (Barium) to oxygen (~80-120 cm^{-1}) and Non-bridging oxygen to metal ion (150 -240 cm^{-1}) bond vibrations.

Figure 2: Transmission spectrum of Optical Filters used while recording the Emission spectra

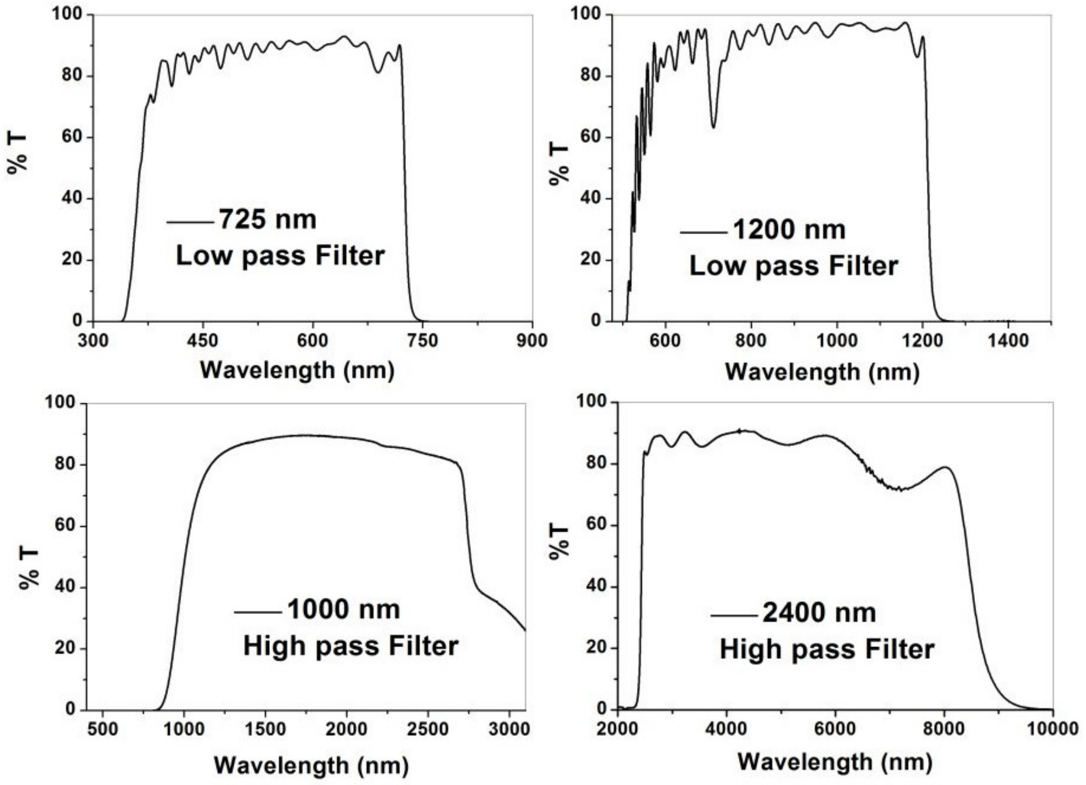

All Filters were procured from EDMOUNT Optics, USA.

To avoid higher order harmonic peaks associated with the excitation and emission wavelengths, following filters were used accordingly.

1. 725 Low pass Filter used at the excitation channel when excitation wavelength are 464 and 653 nm.
2. 1200 Low pass Filter used at the excitation channel when exciting at 985 nm and 1183 nm.
3. NIR emission spectra have been recoded using suitable low pass filter at excitation channel and 1000 nm high pass filter at emission channel.
4. MIR emission spectra have been recoded using suitable low pass filter at excitation channel and 2400 nm high pass filter at emission channel.

Figure 3: MIR Emission Cross-section spectrum derived from Fuchtbauer-Ladenburg (FL) equation.

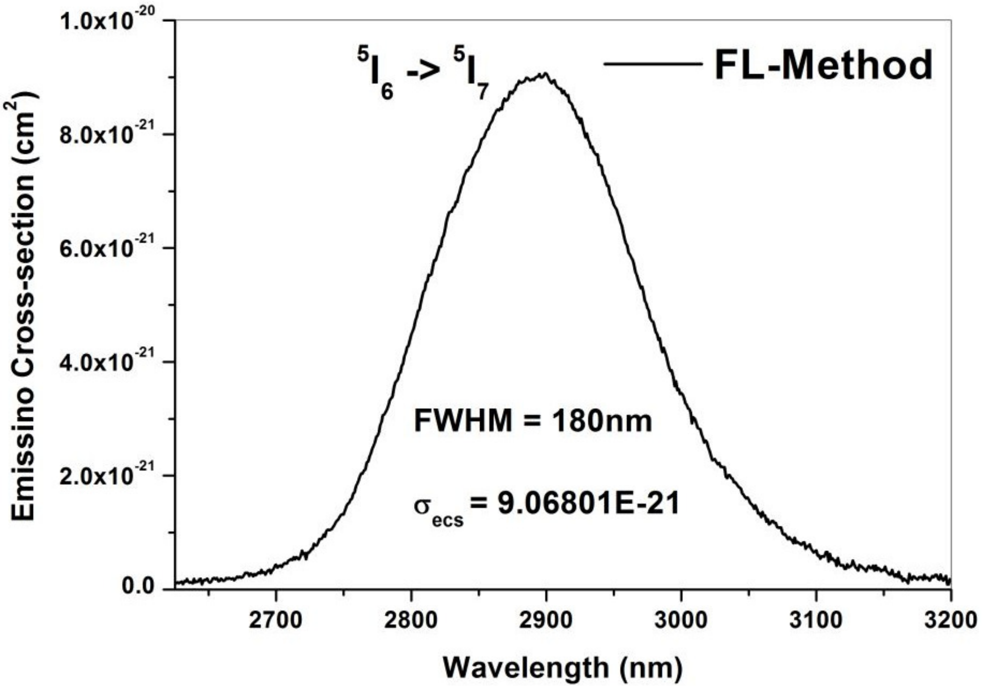

Excited State Absorption (ESA) cross-section Calculation:

The ESA cross-section values have been calculated using the formula[1, 2]

$$\sigma_{ESA} = \frac{8\pi^2 e^2 (n+2)^2}{27hc(2J+1)n} S_{ed}$$

Where S_{ed} is the electric dipole line transition strength (calculated from J-O analysis). σ_{ESA} for $^5I_7 \rightarrow {}^5I_5$ transition under Ho^{3+}: 1836 nm excitation is calculated as 1.313×10^{-22} cm^2, for $^5I_7 \rightarrow {}^5F_5$ transition under Yb^{3+}: 985 nm and Ho^{3+}: 1183 nm excitations are calculated as $7.709\text{x}10^{-23}$ and 5.607×10^{-23} cm^2 respectively. ESA under Ho^{3+}:1836 nm is much efficient because of its high absorption cross section and strong electric dipole line strength compared to other excitations.

Ref:

1. P. Le BouLanger, J-L. Doualan, S. Girard, J. Margerie, R. Moncorge, Phy. Rev. B. **60**, 11380 (1999)
2. B. Di. Bartolo, X. Chen, Advances In Energy Transfer Process, World Scientific, (1999)

Figure 4: Measured Decay curves of Ho^{3+}: $^5I_6 \rightarrow ^5I_8$ (1.2µm) transition

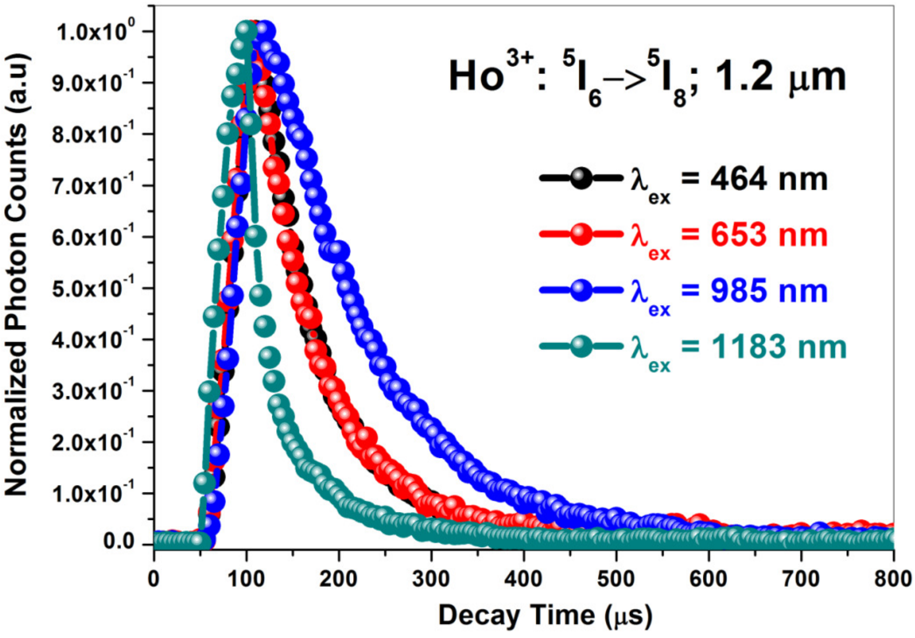

Figure 4 present the as measured decay curves for the 1.2µm emission corresponding to the transition Ho^{3+}: $^5I_6 \rightarrow ^5I_8$ under different excitation wavelengths.

Figure 5: FTIR absorption spectrum of Yb^{3+}/Ho^{3+} co-doped tellurite glass

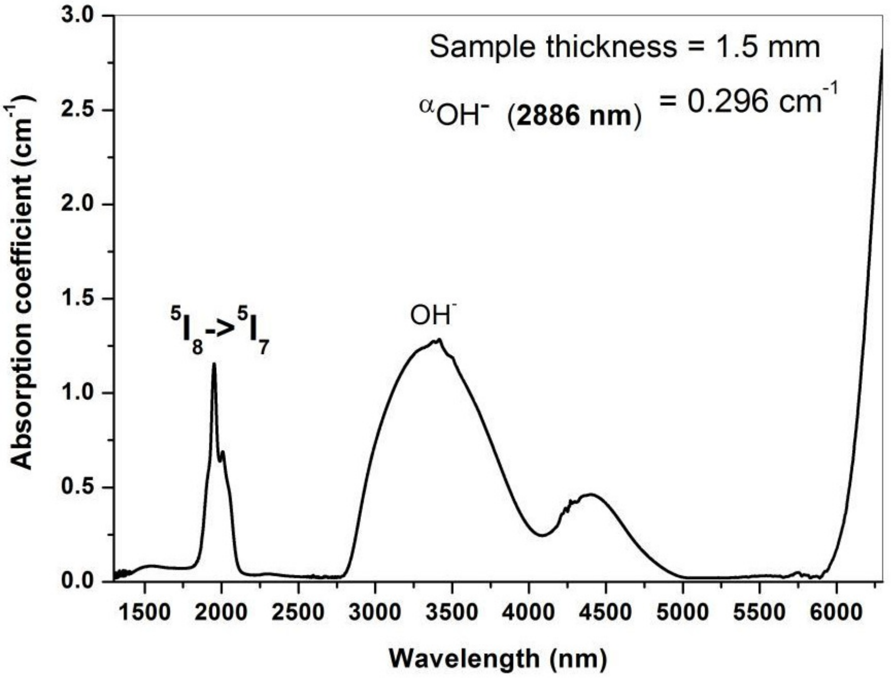

Figure 5presents FTIR absorption spectrum of Ho^{3+}/Yb^{3+} co-doped sample which also indicates OH^- content in the glass determined at MIR emission peak wavelength of Ho^{3+} ions. It can be noted from Fig 4 that, the ~2.9µm emission energy is well above the absorption energy of OH^- molecules in this tellurite glass host which helped to perceive MIR emission transition from Ho^{3+} ions at ~2.9µm. However, despite the strong resonant energy transfer from Yb^{3+}: $^2F_{5/2}$ to Ho^{3+}: $^5I_5$ level, the ~4µm emission (originating from Ho^{3+}: $^5I_5$ level) has not been observed since the energy of this ~4µm emission is well short of the strong absorption band of OH^- molecules as depicted in Fig 4. Further, the OH^- content in the glass is comparable or even less than other tellurite glass where MIR emissions from Dy^{3+} ions were reported very recently (ref.15 of manuscript).
